# Poly[[[μ-*trans*-1,2-bis­(pyridin-4-yl)ethene-κ^2^ *N*:*N*′]-μ-iodido-copper(I)]–*trans*-1,2-bis­(pyridin-4-yl)ethene (1/0.25)]

**DOI:** 10.1107/S2414314620009980

**Published:** 2020-07-24

**Authors:** Jessica L. Hoffman, Jessie E. Akhigbe, Eric W. Reinheimer, Bradley W. Smucker

**Affiliations:** a Austin College, 900 N Grand, Sherman, TX 75090, USA; b Rigaku Oxford Diffraction, 9009 New Trails Dr., The Woodlands, TX 77381, USA; Benemérita Universidad Autónoma de Puebla, México

**Keywords:** crystal structure, coordination polymer, CuI dimer, copper

## Abstract

The title compound, {[CuI(bpe)]·0.25(bpe)}_*n*_, was synthesized similarly to (CuI)_2_(bpe) [Neal *et al.* (2019 ▸). *IUCrData*, **4**, x190122] with red crystals grown from aceto­nitrile solutions of CuI and the bpe ligand [bpe = 1,2-bis­(pyridin-4-yl)ethene, C_12_H_10_N_2_]. The structure of the title compound is a type 1 complex in the Graham nomenclature [Graham *et al.* (2000 ▸). *Inorg. Chem*. **39**, 5121–5132], having rhombic dimers of Cu_2_I_2_ that are bridged by two bpe ligands, to form oligomeric ribbons arranged as stairsteps. The step height is 2.8072 (11) Å, which is the Cu—I^i^ distance of the dimer [symmetry code (i): 1 − *x*, 2 − *y*, 1 − *z*]. The resulting polymer displays a two-dimensional honeycomb framework along the (01  ) plane, and disordered free bpe mol­ecules fill the voids in the crystal.

## Abstract 2

In the title polymer, oligomeric stairsteps are composed of rhombus dimers of Cu_2_I_2_ that are bridged by 1,2-bis­(pyridin-4-yl)ethene ligands.

## Graphical abstract

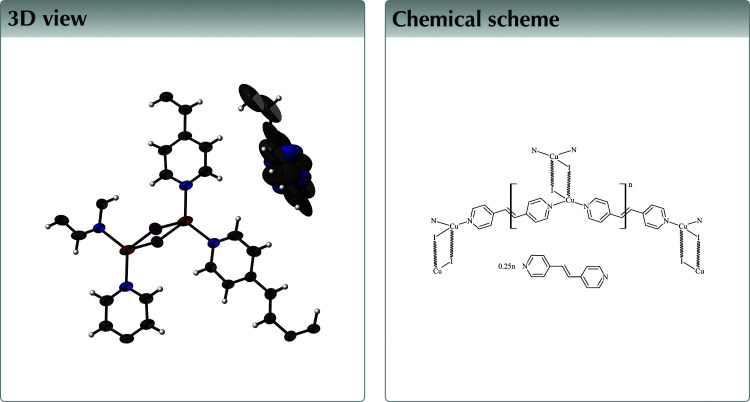

## Structure description

**Figure 1:**
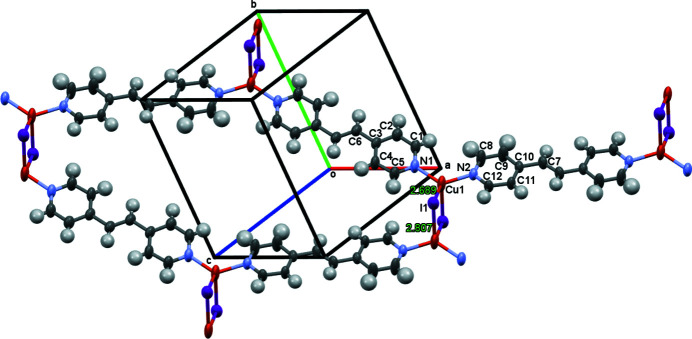
Displacement ellipsoid plot (50% probability level) of all non-H atoms for the oligomeric ribbons of Cu_2_I_2_ dimers bridged by bpe and arranged as stairsteps with 2.8072 (11) Å height (the Cu—I^i^ distance). Cu—I and Cu—I^i^ distances are shown. Guest bpe mol­ecule are omitted for clarity.

The structure of the title compound contains discrete rhombic dimers of Cu_2_I_2_, where the Cu—I distance is 2.6891 (9) Å, the distance across the dimer (Cu—I^i^ distance) is 2.8072 (11) Å, and the Cu⋯Cu^i^ separation is 3.544 (1) Å [symmetry code (i): 1 − *x*, 2 − *y*, 1 − *z*]. The approximately tetra­hedral geometry around the Cu^I^ atoms has an N—Cu—N angle of 127.33 (17)° and I—Cu—I^i^ angle of 99.74 (3)°. Each bpe ligand connects two copper(I) atoms to form oligomeric zigzag ribbons of CuI(bpe), which can be classified as a type 1 complex (Graham *et al.*, 2000 ▸), where bpe is 1,2-bis­(pyridin-4-yl)ethene. These ribbons are arranged as stairsteps with each stair resulting from the Cu_2_I_2_ dimer, hence the step height is 2.8072 (11) Å (the Cu—I^i^ distance, Fig. 1 ▸). This packing is quite different from the analogous CuI(4,4′-bipyrid­yl) complex, where tetra­meric units, composed of two Cu_2_I_2_ dimers bridged by two 4,4′-bipyridyl ligands, are linked by additional 4,4′-bipyridyl ligands to form inter­penetrating hexa­gonal honeycomb sheets (Blake *et al.*, 1999 ▸).

The title compound is quite similar to structures of [CuI(bpe)] containing guest aniline or *p*-toluidine mol­ecules (Yang *et al.*, 2011 ▸), except that it contains a bpe mol­ecule, which is disordered over two inversion centers, with occupancy of 0.25. In attempts at identifying this guest mol­ecule, we considered bpe and aceto­nitrile (crystallization solvent). Refinements on either mol­ecule required substantial restraints and yielded unsatisfactory results. The final model for both, however, gave normal displacement parameters. A lack of C≡N vibrations in the IR spectra of crystals ultimately led towards assigning the guest as a disordered bpe mol­ecule. The use of *SQUEEZE* (Spek, 2015 ▸) also seemed less ideal as the position of the guest was evident in difference maps.

## Synthesis and crystallization

The title compound was synthesized using the same procedure as reported in the synthesis of polymeric [(CuI)_2_(bpe)] (Neal *et al.*, 2019 ▸; Parmeggiani & Sacchetti, 2012 ▸). Red crystals were grown by layering an aceto­nitrile solution containing freshly prepared CuI, ascorbic acid and KI with another aceto­nitrile solution containing bpe in a thin tube. The concentration of bpe in this tube is inferred to be greater than the concentration of CuI to afford the red type 1 complexes of [CuI(bpe)] rather than the aforementioned complexes of [(CuI)_2_(bpe)], which are type 2 (Graham *et al.*, 2000 ▸). Similar structures of [(CuI)(bpe)] were reported with guest aniline or *p*-toluidine mol­ecules but were made from solvothermal reactions (Yang *et al.*, 2011 ▸).

## Refinement

Details of the crystal data, data collection, and structure refinement are summarized in Table 1 ▸. One-half of the guest bpe mol­ecule is placed close to an inversion center, and its occupancy was fixed to 0.5. As a result, the amount of guest bpe for each CuI(bpe) monomer is 0.25. The geometry of the disordered guest mol­ecule was fully restrained using 1,2 and 1,3 distances from a known target. This mol­ecule was also restrained to be flat, with standard deviation of 0.1 Å^3^, while displacement parameters were restrained, with effective standard deviation of 0.1 Å^2^ to approximate an isotropic behaviour. Finally, rigid bond restraints were applied to the guest bpe mol­ecule (Sheldrick, 2015*b* ▸).

## Supplementary Material

Crystal structure: contains datablock(s) I, global. DOI: 10.1107/S2414314620009980/bh4052sup1.cif

Structure factors: contains datablock(s) I. DOI: 10.1107/S2414314620009980/bh4052Isup2.hkl

Supporting information file. DOI: 10.1107/S2414314620009980/bh4052Isup3.mol

CCDC reference: 2017730

Additional supporting information:  crystallographic information; 3D view; checkCIF report

## Figures and Tables

**Table 1:** Experimental details

Crystal data	
Chemical formula	[CuI(C_12_H_10_N_2_)·0.25C_12_H_10_N_2_
*M*_r_	418.21
Crystal system, space group	Triclinic, *P* 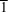
Temperature (K)	293
*a*, *b*, *c* (Å)	7.9004 (2), 10.4260 (3), 10.5078 (3)
α, β, γ (°)	99.903 (2), 104.930 (2), 110.061 (3)
*V* (Å^3^)	752.55 (4)
*Z*	2
Radiation type	Mo *K*α
μ (mm^−1^)	3.49
Crystal size (mm)	0.14 × 0.10 × 0.06

Data collection	
Diffractometer	Rigaku XtaLAB Mini II
Absorption correction	Multi-scan (*CrysAlis PRO*; Rigaku OD, 2019 ▸)
*T*_min_, *T*_max_	0.838, 1.000
No. of measured, independent and observed [*I* > 2σ(*I*)] reflections	16024, 2683, 2021
*R*_int_	0.033
(sin θ/λ)_max_ (Å^−1^)	0.597

Refinement	
*R*[*F* ^2^ > 2σ(*F* ^2^)], *wR*(*F* ^2^), *S*	0.035, 0.091, 1.03
No. of reflections	2683
No. of parameters	200
No. of restraints	87
H-atom treatment	H-atom parameters constrained
Δρ_max_, Δρ_min_ (e Å^−3^)	0.60, −1.01

Computer programs: *CrysAlis PRO* (Rigaku OD, 2019 ▸), *SHELXT2018/2* (Sheldrick, 2015*a* ▸), *SHELXL2018/3* (Sheldrick, 2015*b* ▸), *Mercury* (Macrae *et al.*, 2020 ▸) and *publCIF* (Westrip, 2010 ▸).

## full crystallographic data

### Crystal data

**Table:**

[CuI(C_12_H_10_N_2_)·0.25C_12_H_10_N_2_	*Z* = 2
*M**_r_* = 418.21	*F*(000) = 404
Triclinic, *P*1	*D*_x_ = 1.846 Mg m^−^^3^
*a* = 7.9004 (2) Å	Mo *K*α radiation, λ = 0.71073 Å
*b* = 10.4260 (3) Å	Cell parameters from 6736 reflections
*c* = 10.5078 (3) Å	θ = 2.1–24.7°
α = 99.903 (2)°	µ = 3.49 mm^−^^1^
β = 104.930 (2)°	*T* = 293 K
γ = 110.061 (3)°	Block, red
*V* = 752.55 (4) Å^3^	0.14 × 0.10 × 0.06 mm

### Data collection

**Table:**

Rigaku XtaLAB Mini II diffractometer	2683 independent reflections
Radiation source: fine-focus sealed X-ray tube, Rigaku (Mo) X-ray Source	2021 reflections with *I* > 2σ(*I*)
Detector resolution: 10.0000 pixels mm^-1^	*R*_int_ = 0.033
ω scans	θ_max_ = 25.1°, θ_min_ = 2.1°
Absorption correction: multi-scan (CrysAlis Pro; Rigaku OD, 2019)	*h* = −9→9
*T*_min_ = 0.838, *T*_max_ = 1.000	*k* = −12→12
16024 measured reflections	*l* = −12→12

### Refinement

**Table:**

Refinement on *F*^2^	Secondary atom site location: difference Fourier map
Least-squares matrix: full	Hydrogen site location: mixed
*R*[*F*^2^ > 2σ(*F*^2^)] = 0.035	H-atom parameters constrained
*wR*(*F*^2^) = 0.091	*w* = 1/[σ^2^(*F*_o_^2^) + (0.0389*P*)^2^ + 1.4132*P*] where *P* = (*F*_o_^2^ + 2*F*_c_^2^)/3
*S* = 1.03	(Δ/σ)_max_ = 0.001
2683 reflections	Δρ_max_ = 0.60 e Å^−^^3^
200 parameters	Δρ_min_ = −1.01 e Å^−^^3^
87 restraints	Extinction correction: SHELXL-2018/3 (Sheldrick, 2015b), Fc^*^=kFc[1+0.001xFc^2^λ^3^/sin(2θ)]^-1/4^
0 constraints	Extinction coefficient: 0.0018 (8)
Primary atom site location: dual	

### Fractional atomic coordinates and isotropic or equivalent isotropic displacement parameters (Å^2^)

**Table:**

	*x*	*y*	*z*	*U*_iso_*/*U*_eq_	Occ. (<1)
I1	0.26536 (6)	0.84672 (5)	0.33204 (4)	0.06679 (19)	
Cu1	0.47272 (12)	0.86742 (10)	0.58757 (8)	0.0777 (3)	
N1	0.6174 (6)	0.7452 (5)	0.5719 (5)	0.0605 (12)	
N2	0.3214 (6)	0.8771 (5)	0.7122 (4)	0.0515 (10)	
C1	0.6526 (9)	0.6715 (7)	0.6588 (6)	0.0683 (16)	
H1	0.603438	0.674885	0.730152	0.082*	
C2	0.7563 (8)	0.5909 (6)	0.6505 (6)	0.0657 (16)	
H2	0.773735	0.540343	0.713949	0.079*	
C3	0.8346 (8)	0.5847 (6)	0.5482 (6)	0.0572 (14)	
C4	0.7964 (9)	0.6579 (7)	0.4552 (7)	0.0743 (18)	
H4	0.843618	0.655522	0.382777	0.089*	
C5	0.6875 (9)	0.7351 (7)	0.4697 (7)	0.0752 (18)	
H5	0.661750	0.782544	0.404776	0.090*	
C6	0.9505 (8)	0.5005 (6)	0.5415 (6)	0.0614 (15)	
H6	0.953705	0.443793	0.600578	0.074*	
C7	0.0594 (8)	0.9694 (6)	1.0045 (5)	0.0578 (14)	
H7	0.093427	0.942977	1.084213	0.069*	
C8	0.3474 (10)	0.8408 (8)	0.8268 (6)	0.090 (2)	
H8	0.427047	0.792999	0.844460	0.108*	
C9	0.2652 (10)	0.8686 (8)	0.9223 (6)	0.089 (2)	
H9	0.290839	0.840143	1.001801	0.107*	
C10	0.1457 (7)	0.9378 (5)	0.9018 (5)	0.0470 (12)	
C11	0.1145 (8)	0.9735 (6)	0.7811 (6)	0.0625 (15)	
H11	0.034006	1.020025	0.760252	0.075*	
C12	0.2020 (8)	0.9406 (6)	0.6906 (6)	0.0643 (16)	
H12	0.175498	0.964583	0.608684	0.077*	
C13	0.9182 (15)	0.4586 (15)	1.002 (3)	0.207 (11)	0.5
H13	0.901139	0.363730	1.005612	0.248*	0.5
C14	0.7563 (17)	0.4951 (16)	0.9907 (15)	0.157 (8)	0.5
C15	0.7591 (17)	0.6220 (15)	1.0534 (17)	0.173 (9)	0.5
H15	0.873366	0.708859	1.090084	0.207*	0.5
C16	0.5871 (15)	0.6190 (9)	1.0550 (15)	0.265 (11)	
H16	0.587357	0.707340	1.100402	0.318*	0.5
H	0.298137	0.295930	0.924152	0.318*	0.5
N17	0.4132 (18)	0.5088 (14)	1.002 (3)	0.268 (16)	0.5
C18	0.5760 (17)	0.3832 (16)	0.926 (2)	0.161 (8)	0.5
H18	0.568167	0.300260	0.863881	0.193*	0.5

### Atomic displacement parameters (Å^2^)

**Table:**

	*U*^11^	*U*^22^	*U*^33^	*U*^12^	*U*^13^	*U*^23^
I1	0.0821 (3)	0.0961 (3)	0.0635 (3)	0.0621 (3)	0.0395 (2)	0.0464 (2)
Cu1	0.0954 (6)	0.1227 (7)	0.0726 (5)	0.0872 (6)	0.0535 (4)	0.0401 (5)
N1	0.068 (3)	0.084 (3)	0.065 (3)	0.056 (3)	0.039 (2)	0.030 (2)
N2	0.061 (3)	0.068 (3)	0.053 (2)	0.045 (2)	0.033 (2)	0.025 (2)
C1	0.081 (4)	0.100 (5)	0.067 (4)	0.067 (4)	0.043 (3)	0.039 (3)
C2	0.075 (4)	0.084 (4)	0.076 (4)	0.057 (4)	0.041 (3)	0.039 (3)
C3	0.055 (3)	0.064 (3)	0.077 (4)	0.043 (3)	0.032 (3)	0.028 (3)
C4	0.091 (4)	0.104 (5)	0.085 (4)	0.076 (4)	0.059 (4)	0.046 (4)
C5	0.098 (5)	0.106 (5)	0.082 (4)	0.081 (4)	0.058 (4)	0.052 (4)
C6	0.064 (4)	0.064 (3)	0.086 (4)	0.044 (3)	0.041 (3)	0.034 (3)
C7	0.073 (4)	0.088 (4)	0.047 (3)	0.055 (3)	0.036 (3)	0.034 (3)
C8	0.123 (6)	0.162 (7)	0.073 (4)	0.123 (6)	0.062 (4)	0.064 (4)
C9	0.129 (6)	0.160 (7)	0.065 (4)	0.122 (6)	0.061 (4)	0.070 (4)
C10	0.050 (3)	0.063 (3)	0.046 (3)	0.035 (3)	0.026 (2)	0.021 (2)
C11	0.082 (4)	0.093 (4)	0.067 (3)	0.071 (4)	0.050 (3)	0.046 (3)
C12	0.094 (4)	0.094 (4)	0.064 (3)	0.073 (4)	0.054 (3)	0.050 (3)
C13	0.23 (2)	0.15 (2)	0.20 (2)	0.062 (19)	0.00 (2)	0.10 (2)
C14	0.27 (2)	0.124 (12)	0.066 (11)	0.082 (13)	0.017 (13)	0.050 (10)
C15	0.162 (16)	0.099 (11)	0.17 (2)	0.014 (12)	−0.024 (16)	0.035 (12)
C16	0.229 (17)	0.209 (17)	0.233 (19)	0.099 (14)	−0.031 (15)	−0.085 (14)
N17	0.192 (18)	0.28 (3)	0.23 (3)	0.106 (17)	0.02 (2)	−0.12 (2)
C18	0.24 (2)	0.124 (14)	0.120 (17)	0.089 (14)	0.042 (17)	0.043 (12)

### Geometric parameters (Å, º)

**Table:**

I1—Cu1	2.6891 (9)	C9—C10	1.370 (7)
I1—Cu1^i^	2.8072 (11)	C9—H9	0.9300
Cu1—N1	1.996 (4)	C10—C11	1.368 (7)
Cu1—N2	2.000 (4)	C11—C12	1.374 (6)
N1—C1	1.326 (7)	C11—H11	0.9300
N1—C5	1.333 (6)	C12—H12	0.9300
N2—C8	1.311 (7)	C13—C13^iv^	1.300 (10)
N2—C12	1.322 (6)	C13—C14	1.437 (9)
C1—C2	1.367 (7)	C13—H13	0.9609
C1—H1	0.9300	C14—N17^v^	1.348 (15)
C2—C3	1.375 (7)	C14—C15	1.363 (9)
C2—H2	0.9300	C14—C18	1.394 (9)
C3—C4	1.376 (8)	C15—C16	1.353 (9)
C3—C6	1.475 (7)	C15—N17^v^	1.45 (2)
C4—C5	1.383 (7)	C15—H15	0.9607
C4—H4	0.9300	C15—H^v^	1.11 (2)
C5—H5	0.9300	C16—C18^v^	1.348 (9)
C6—C6^ii^	1.314 (10)	C16—N17	1.350 (9)
C6—H6	0.9300	C16—N17^v^	1.362 (9)
C7—C7^iii^	1.297 (9)	C16—H16	0.9608
C7—C10	1.466 (6)	C16—H^v^	0.967 (18)
C7—H7	0.9300	N17—C18^v^	1.21 (2)
C8—C9	1.370 (7)	N17—N17^v^	1.45 (2)
C8—H8	0.9300	C18—H18	0.9607
			
Cu1—I1—Cu1^i^	80.26 (3)	N17^v^—C14—C15	64.6 (10)
N1—Cu1—N2	127.33 (17)	N17^v^—C14—C18	52.3 (10)
N1—Cu1—I1	108.03 (13)	C15—C14—C18	116.2 (10)
N2—Cu1—I1	109.45 (12)	N17^v^—C14—C13	160.3 (17)
N1—Cu1—I1^i^	107.96 (15)	C15—C14—C13	126.9 (13)
N2—Cu1—I1^i^	100.67 (13)	C18—C14—C13	116.2 (11)
I1—Cu1—I1^i^	99.74 (3)	C16—C15—C14	115.2 (10)
C1—N1—C5	115.8 (4)	C16—C15—N17^v^	58.1 (6)
C1—N1—Cu1	124.3 (3)	C14—C15—N17^v^	57.2 (8)
C5—N1—Cu1	119.9 (4)	C16—C15—H15	121.9
C8—N2—C12	115.1 (4)	C14—C15—H15	122.8
C8—N2—Cu1	124.8 (3)	N17^v^—C15—H15	179.9
C12—N2—Cu1	119.5 (3)	C16—C15—H^v^	44.9 (10)
N1—C1—C2	124.4 (5)	C14—C15—H^v^	157.2 (16)
N1—C1—H1	117.8	N17^v^—C15—H^v^	102.3 (13)
C2—C1—H1	117.8	H15—C15—H^v^	77.6
C1—C2—C3	119.9 (5)	C18^v^—C16—N17	53.2 (10)
C1—C2—H2	120.1	C18^v^—C16—C15	172.4 (16)
C3—C2—H2	120.1	N17—C16—C15	129.2 (11)
C2—C3—C4	116.6 (4)	C18^v^—C16—N17^v^	116.9 (11)
C2—C3—C6	119.8 (5)	N17—C16—N17^v^	64.9 (11)
C4—C3—C6	123.5 (5)	C15—C16—N17^v^	64.5 (9)
C3—C4—C5	119.8 (5)	C18^v^—C16—H16	62.0
C3—C4—H4	120.1	N17—C16—H16	114.6
C5—C4—H4	120.1	C15—C16—H16	116.1
N1—C5—C4	123.5 (5)	N17^v^—C16—H16	176.3
N1—C5—H5	118.3	C18^v^—C16—H^v^	125.1 (15)
C4—C5—H5	118.3	N17—C16—H^v^	168 (3)
C6^ii^—C6—C3	125.1 (7)	C15—C16—H^v^	54.4 (13)
C6^ii^—C6—H6	117.4	N17^v^—C16—H^v^	118.0 (18)
C3—C6—H6	117.4	H16—C16—H^v^	63.3
C7^iii^—C7—C10	126.7 (6)	C18^v^—N17—C14^v^	65.8 (8)
C7^iii^—C7—H7	116.7	C18^v^—N17—C16	63.3 (7)
C10—C7—H7	116.7	C14^v^—N17—C16	128.9 (12)
N2—C8—C9	124.4 (5)	C18^v^—N17—C16^v^	168 (3)
N2—C8—H8	117.8	C14^v^—N17—C16^v^	115.6 (11)
C9—C8—H8	117.8	C16—N17—C16^v^	115.1 (11)
C10—C9—C8	120.6 (5)	C18^v^—N17—C15^v^	123.2 (12)
C10—C9—H9	119.7	C14^v^—N17—C15^v^	58.2 (7)
C8—C9—H9	119.7	C16—N17—C15^v^	172.1 (15)
C11—C10—C9	115.5 (4)	C16^v^—N17—C15^v^	57.5 (7)
C11—C10—C7	123.8 (4)	C18^v^—N17—N17^v^	120.1 (14)
C9—C10—C7	120.8 (4)	C14^v^—N17—N17^v^	171 (3)
C10—C11—C12	120.1 (5)	C16—N17—N17^v^	58.0 (7)
C10—C11—H11	120.0	C16^v^—N17—N17^v^	57.1 (7)
C12—C11—H11	120.0	C15^v^—N17—N17^v^	114.5 (11)
N2—C12—C11	124.4 (5)	N17^v^—C18—C16^v^	63.5 (7)
N2—C12—H12	117.8	N17^v^—C18—C14	61.9 (9)
C11—C12—H12	117.8	C16^v^—C18—C14	125.2 (12)
C13^iv^—C13—C14	124.7 (16)	N17^v^—C18—H18	176.4
C13^iv^—C13—H13	117.5	C16^v^—C18—H18	117.6
C14—C13—H13	117.6	C14—C18—H18	117.2
			
C5—N1—C1—C2	1.2 (10)	C13^iv^—C13—C14—C15	−43 (5)
Cu1—N1—C1—C2	−178.1 (5)	C13^iv^—C13—C14—C18	147 (3)
N1—C1—C2—C3	1.1 (11)	N17^v^—C14—C15—C16	−2.9 (18)
C1—C2—C3—C4	−2.4 (9)	C18—C14—C15—C16	5.8 (16)
C1—C2—C3—C6	178.5 (6)	C13—C14—C15—C16	−164.1 (18)
C2—C3—C4—C5	1.4 (10)	C18—C14—C15—N17^v^	8.7 (18)
C6—C3—C4—C5	−179.5 (6)	C13—C14—C15—N17^v^	−161 (2)
C1—N1—C5—C4	−2.3 (10)	C14—C15—C16—N17	−1 (2)
Cu1—N1—C5—C4	177.0 (5)	N17^v^—C15—C16—N17	−4 (3)
C3—C4—C5—N1	1.0 (11)	C14—C15—C16—N17^v^	2.9 (18)
C2—C3—C6—C6^ii^	−172.0 (8)	C15—C16—N17—C18^v^	171 (2)
C4—C3—C6—C6^ii^	8.9 (12)	N17^v^—C16—N17—C18^v^	167 (3)
C12—N2—C8—C9	−2.1 (11)	C18^v^—C16—N17—C14^v^	6 (2)
Cu1—N2—C8—C9	169.2 (6)	C15—C16—N17—C14^v^	177 (2)
N2—C8—C9—C10	0.4 (13)	N17^v^—C16—N17—C14^v^	173 (5)
C8—C9—C10—C11	0.9 (11)	C18^v^—C16—N17—C16^v^	−167 (3)
C8—C9—C10—C7	−179.2 (7)	C15—C16—N17—C16^v^	4 (3)
C7^iii^—C7—C10—C11	1.9 (12)	N17^v^—C16—N17—C16^v^	0.001 (1)
C7^iii^—C7—C10—C9	−178.0 (8)	C18^v^—C16—N17—N17^v^	−167 (3)
C9—C10—C11—C12	−0.5 (9)	C15—C16—N17—N17^v^	4 (3)
C7—C10—C11—C12	179.6 (6)	C15—C14—C18—N17^v^	−10 (2)
C8—N2—C12—C11	2.6 (10)	C13—C14—C18—N17^v^	161 (2)
Cu1—N2—C12—C11	−169.2 (5)	N17^v^—C14—C18—C16^v^	−5 (2)
C10—C11—C12—N2	−1.3 (10)	C15—C14—C18—C16^v^	−15 (3)
C13^iv^—C13—C14—N17^v^	−163 (4)	C13—C14—C18—C16^v^	156 (2)

Symmetry codes: (i) −*x*+1, −*y*+2, −*z*+1; (ii) −*x*+2, −*y*+1, −*z*+1; (iii) −*x*, −*y*+2, −*z*+2; (iv) −*x*+2, −*y*+1, −*z*+2; (v) −*x*+1, −*y*+1, −*z*+2.
